# The association between *FTO* genotype with macronutrients and calorie intake in overweight adults

**DOI:** 10.1186/s12944-020-01372-x

**Published:** 2020-08-26

**Authors:** Mahsa Mehrdad, Saeid Doaei, Maryam Gholamalizadeh, Mohammad Hassan Eftekhari

**Affiliations:** 1 Department of Clinical Nutrition, School of Nutrition and food Sciences, Shiraz University of Medical Sciences, Shiraz, Iran; 2 Cancer Research Center, Shahid Beheshti University of Medical Sciences, Tehran, Iran; 3 Research Center of Health and Environment, Guilan University of Medical Sciences, Rasht, Iran; 4 Student research committee, Cancer Research Center, Shahid Beheshti University of Medical Sciences, Tehran, Iran

**Keywords:** *FTO*, Polymorphism, Genotype, Macronutrient, Carbohydrate, Protein, Fat, Fiber

## Abstract

**Background:**

Dietary macronutrients may indirectly affect body weight through their interactions with the fat mass and obesity associated (*FTO*) gene. This study aimed to investigate the association between *FTO* gene rs9939609 polymorphism with macronutrients intake in overweight adults.

**Methods:**

This study was carried out on 196 overweight adults of Shiraz, Iran. Dietary intake was assessed using a validated 168-item semi-quantitative food frequency questionnaire (FFQ). The *FTO* gene was genotyped for rs9939609 polymorphism. The association between dietary macronutrients and the *FTO* genotype were assessed using linear regression after adjustments for sex, age, physical activity, and the serum levels of triglycerides, fasting blood sugar (FBS), and low density lipoprotein (LDL).

**Results:**

The higher intake of carbohydrates (*P* < 0.001), fat (*P* = 0.009), and calorie (*P* = 0.001) were significantly associated with rs9939609 AA genotype (*P* = 0.001). Carriers of the AA genotype of rs9939609 had significantly higher calorie, fat, and carbohydrate intake than the carriers of the TT genotype after adjusting for age and sex (*P* = 0.019, *P* = 0.010 and *P* = 0.001, respectively). Further adjustments for physical activity, TG, LDL, and FBS did not change these results.

**Conclusion:**

The amounts of dietary calorie, carbohydrate, and fat intake were associated with *FTO* genotype. Further studies are warranted to confirm these associations and to identify the underlying mechanisms.

## Introduction

The prevalence of obesity as a health-related problem has been dramatically increased in both developed and developing countries [1, 2]. More than 30% of adults’ population of the United States are obese [3]. Obesity is associated with other chronic diseases such as cancer, hypertension, dyslipidemia, cardiovascular disease, type 2 diabetes, and psychological disorders [4]. Obesity is a multifactorial disorder caused by genetics, lifestyle, and environmental factors [5, 6].

The role of some genes in obesity has been reported in many studies [7–9]. The fat mass and obesity associated (*FTO)* gene is located on the chromosome region 16q12.2 and was reported to be strongly associated with obesity [7, 8]. The *FTO* gene is widely expressed in several tissues such as brain, visceral fat, liver, and hypothalamus. Several studies reported that *FTO* genotype has a strong association with body mass index (BMI) and obesity [8, 9]. *FTO* rs9939609 polymorphism is associated with the increased risk of obesity. People with rs9939609 *FTO* variant alleles (homozygous = AA and heterozygous = AT) are predisposed to greater adiposity than are those with 2 wild-type alleles (TT). The minor allele frequency of rs9939609 is much different based on ethnicity, i.e. it is about 0.449, 0.122, 0.178, and 0.517 in European, Chinese, Japanese, and African populations, respectively [10].

*FTO* gene has an important role in regulation of food intake, energy balance, appetite, and basal metabolic rate (BMR) [11, 12]. Polymorphisms in the intron regions of *FTO* gene may act as a regulator of other genes such as Iroquois homeobox 3 (*IRX3*) and obesity-associated single nucleotide polymorphisms of FTO were associated with expression of *IRX3*, but not *FTO*, in human brains [11]. On the other hand, *FTO* genotypes may influence the association of dietary macronutrients with Body Mass Index (BMI), body weight, food intake, energy balance, appetite, and hormone secretion [12–14]. Dietary macronutrients including carbohydrate, fat, and protein, as the main sources of energy, play key roles in regulation of body weight and BMI [14, 15]. However, the effects of polymorphisms in obesity-related genes on the amount of macronutrients’ intake is not clear. So, this study aimed to investigate the interactions between the amount of dietary carbohydrate, protein, and fat with the *FTO* genotype in overweight adults.

## Methodology

This study was carried out from September 2016 to October 2017 on 199 randomly selected participants referred to the Shohadaye Valfajr health center, Shiraz, Iran. Participants were overweight adults aged 20 to 45 years with BMI between 25 to 29.9 kg/m^2^. The Inclusion criteria was defined as: healthy people with overweight, willingness to participation in the study, not participating in a weight management programs during two past months, and no weight loss greater than 5% over the last 3 months. Participants with alcohol or drugs addiction (*n* = 1), smoking, certain weight-related diseases (including specific psychological or neurological disorders, insulin resistance, thyroid diseases, liver diseases, renal failure, and infectious diseases) (*n* = 1), and pregnant or lactating women (*n* = 1) were excluded. Thus, the final number of participants in this study was 196. All participants signed a consent form before participation in the study.

### Anthropometric measures

The height of the participants was measured with a calibrated tape line fastened to a wall and without shoes with a precision of 0.5 cm. A bio impedance analysis (BIA) scale (BC-418, Tanita Cooperation, Tokyo, Japan) was then used to measure anthropometric indices such as BMI, skeletal muscle percentage (SM%), body fat (BF), skeletal muscle (SM) and body fat percentage (BF%) after entering their height, age, and gender.

### Genotyping

DNA was extracted from whole peripheral blood sample using the DNA extraction kit (Cinnagen Company, Tehran, Iran and were stored at − 20 °C before genotyping. The concentration of the extracted material was assessed using spectrophotometer by the NanoDrop® ND-1000 UV-Vis Spectrophotometer (Nanodrop technologies, Rockland, USA). *FTO* gene was genotyped for rs9939609 polymorphism via tetra-primer amplification refractory mutation system-polymerase chain reaction (Tetra-ARMS PCR). The sequences of the primers are presented in supplementary file 1.

### Macronutrients’ intake

Usual Macronutrients’ intakes of the participants were assessed using a validated 168-item semi-quantitative food frequency questionnaires (FFQ) [16]. The FFQ was consisted of 168 food items with standard portion sizes commonly consumed by Iranian people. Face-to-face interviews were conducted by a trained dietitian.

Dietary intake was analyzed using the Nutritionist-4 software program which was modified for Iranian foods [17]. Daily intakes of calorie were measured for each person by using the US Department of Agriculture food consumption database, which was modified for Iranian foods.

### Physical activity

A validated international physical activity questionnaire (IPAQ) was used to measure participants’ physical activity [18]. Results obtained from IPAQ were expressed as metabolic equivalents (MET) per minute.

### Laboratory measurement

The levels of serum triglyceride (TG), total cholesterol (TC), high -density lipoprotein (HDL), low-density lipoprotein cholesterol (LDL), and glucose were measured after 12 h of an overnight fasting.

### Statistical analysis

The Shapiro-Wilk normality normality test was used to determine if the quantitative variables had a normal distribution. ANOVA test was used to compare demographic, anthropometric measurements, macronutrients’ intake, and physical activity between different *FTO* genotypes. The post hoc Tukey’s test was then used to identify significant differences of calorie and macronutrients intake between three genotypes. Linear regression was used to adjust the effects of confounders including age, sex, PA, TG, TC, LDL, and FBS. Statistical analyses were performed using SPSS version 23.0 (IBM SPSS, IBM Corp., Chicago, USA). The results were considered statistically significant at *P* < 0.05.

### Ethics approval and consent to participate

This study has been approved by ethics review board of Shiraz University of Medical Sciences (Code: ir.sums.rec.1395.100).

## Results

All data were normally distributed according to the Shapiro-Wilk normality test. Regarding *FTO* rs9939609 genotype, about half of the participants were heterozygote (*n* = 98), about 30% of them were TT wild type (*n* = 60), and about 20% of them were AA homozygote (*n* = 38). The genotype distribution of the study population was in Hardy-Weinberg equilibrium. Significant differences were found in BMI (*P* = 0.047), fat mass (*P* = 0.001), calorie intake (*P* = 0.001), fat intake (*P* = 0.009), and carbohydrate intake (*P* < 0.001) status of three *FTO* genotypes (Table 1). Carriers of the AA genotype of *FTO* rs9939609 polymorphism had significantly higher calorie, fat, and carbohydrate intake than the carriers of the TT genotype (*P* = 0.019, *P* = 0.010, and *P* = 0.000, respectively) (Table 2).

**Table 1:** characteristics of the subjects categorized by *FTO* rs9939609 genotypes (*N* = 196)

Variables	TT (***n*** = 60)	AT (***n*** = 98)	AA (***n*** = 38)	*P* value
**Male sex N(%)**	15 (25)	25 (25.3)	10 (26.3)	0.989
**Age, years mean (SD)**	33.43(±6.461)	32.99(±6.488)	34.08(±5.961)	0.664
**Weight, kg**	72.140(±9.8058)	72.618(±9.1667)	75.262(±9.3294)	0.241
**Height, m mean (SD)**	163.983(±9.8402)	163.980(±9.4112)	165.816(±9.0609)	0.564
**BMI, kg/m2 mean (SD)**	26.7086(±1.10977)	26.9072(±1.03883)	27.2864(±1.33132)	0.047
**Fat Mass, kg mean (SD)**	21.380(±3.9137)	22.160(±3.3318)	24.363(±4.2223)	0.001
**FM, % mean (SD)**	30.0847(±6.07727)	31.0358(±5.94178)	32.7175(±6.23811)	0.112
**FFM, kg mean (SD)**	50.7600(±10.22795)	50.4586(±10.25310)	50.8989(±9.67006)	0.968
**FFM, % mean (SD)**	69.9153(±6.07727)	68.9642(±5.94178)	67.2825(±6.23811)	0.112
**IPAQ, MET/minute mean (SD)**	1462.08(±1391.611)	1134.34(±1580.308)	823.61(±1159.997)	0.100
**Calorie intake, Kcal mean (SD)**	1595 (±844)	1617(±740)	1992 (±762)	0.001
**Fat, g/day mean (SD)**	24.4433(±11.10069)	24.4573(±11.01026)	31.8353(±15.77593)	0.009
**Protein, g/day mean (SD)**	115.63(±45.54972)	117.43(±41.17454)	152.35(±46.89251)	0.189
**Carbohydrate, g/day mean (SD)**	228.32(±173.8147)	231.85(±158.10551)	273.97(±163.29332)	0.001
**Fiber, g/day mean (SD)**	8.0043(±3.60432)	7.6244(±3.15966)	8.4431(±2.50727)	0.621
**FBS, mg/ dL mean (SD)**	86.95(±8.490)	89.18(±9.738)	91.42(±11.568)	0.084
**LDL-C, mg/ dL mean (SD)**	96.90(±20.755)	102.75(±16.100)	103.82(±18.475)	0.088
**HDL-C, mg/ dL mean (SD)**	47.20(±9.950)	42.82(±8.175)	40.87(±6.751)	0.088
**T Chol, mg/ dL mean (SD)**	183.13(±29.514)	192.42(±23.446)	199.50(±25.438)	0.008
**TG, mg/dL mean (SD)**	113.87(±48.315)	118.03(±27.235)	118.74(±29.388)	0.724

Abbreviations: *BMI* body mass index; *HDL* high-density lipoprotein;; *FFM* fat free mass; *IPAQ* International Physical Activity Questionnaire; *LDL* low-density lipoprotein; *T Chol* total cholesterol; *TG* triglycerides; *FBS* fasting blood sugar; *FAT* fat intake; carbohydrate, carbohydrate intake; *Protein* protein intake; *Fiber* fiber intake

**Table 2:** Tukey test for comparison the calorie and macronutrient intake between three genotypes

Variable	TT	AT	***P*** value	AA	***P*** value
**Calorie**	1	−25.17740	0.869	−173.09213	0.019*
**Fat**	1	−0.01397	1.000	−7.39193	0.010*
**Protein**	1	−3.52699	0.991	−45.65037	0.373
**Carbohydrate**	1	−1.80258	0.966	−36.72395	0.001*
**Fiber**	1	0.37984	0.748	−0.43883	0.785

*P-value ⎕0.05

Linear association of *FTO* rs9939609 genotype with the level of macronutrients’ intake (carbohydrate, fat, protein, and fiber) was then assessed after adjustment the effects of confounders. This association remained significant for carbohydrate, calorie and fat intake after adjustment for age and sex (*P* = 0.000, *P* = 0.001, and *P* = 0.009 respectively) (model 1). Further adjustments for physical activity, TG, LDL, and FBS did not change the results (*P* = 0.001, *P* = 0.000, and *P* = 0.019 respectively) (model 2) (Table 3).

**Table 3:** Association of *FTO* genotypes with macronutrients’ intake

		Model 1			Model 2	
variables	R^2^	Beta	P	R^2^	Beta	P
**Calorie**	0.6	0.149	0.001*	0.6	0.162	0.001*
**FAT**	0. 4	0.186	0.009*	0.4	0.172	0.019*
**Protein**	0.5	0.092	0.189	0.5	0. 090	0.219
**Carbohydrate**	0.2	0.250	0.001*	0.2	0.246	0.001*
**Fiber**	0.3	0. 035	0.621	0.4	0.045	0.545

Model 1: adjusted for age and sex;

Model 2: Further adjustments for physical activity, the serum levels of triglycerides, fasting blood sugar (FBS), and low density lipoprotein (LDL)

*P-value < 0.05

## Discussion

The present study evaluated the associations between rs9939609 *FTO* gene polymorphism with calorie, fat, carbohydrate, protein, and fiber intake. The results identified that there was a significant association between *FTO* genotype with calorie, carbohydrate, and fat intake. This association remained significant for calorie, carbohydrate, and fat intake after adjustments for sex, age, physical activity, LDL, HDL, and FBS. In carriers of AA genotype of rs9939609 polymorphism, dietary carbohydrate, fat, and calorie intake were higher than TT carriers. However, the results of recent studies about the association between dietary macronutrients and *FTO* polymorphism were inconsistent [19–28]. Oyeyemi et al. in a case-control study on 103 people with obesity estimated as BMI ≥ 25 and 98 controls identified 354.4 kcal/d more energy intake per risk A allele of rs9939609 [19]. Timpson et al. reported higher calorie and fat intake among rs9939609 AA genotype carriers. They suggest that *FTO* polymorphism may influence on appetite and food intake [20]. Some other studies also reported that carriers of risk allele *FTO* received higher energy intake [21]. Consistent with the results of this study, Daya et al. reported that carriers of AT/AA genotype had higher fat intake (1.40 times) and had higher risk of obesity compared with TT genotype [22]. The *FTO* variants were reported to be associated with intake of energy-dense foods such as fat-rich foods [23]. *FTO* gene variants played important roles in appetite regulation, food intake, tendency to choose energy-dense food (high fat and high carbohydrate diet) [24]. The carriers of A allele *FTO* rs9939609 had energy-dense food choices, higher body weight, and overeating behaviors [25]. On the other hand, Qi et al. in a cross-sectional study on white population (*n* = 154,439) found a lower energy intake per A risk allele (ß = − 7.2 kcal/d) [26]. Another study found no association between a high-fat diet and a high carbohydrate diet with the *FTO* gene in rats [27]. Drabsch et al. in a systematic review reported that there is no consistent evidence that the *FTO* gene SNPs are associated with total energy, carbohydrate, and fat intakes [28]. The cause of this discrepancy between the studies remained unclear. However, the relationship between *FTO* genotype and dietary intake seems to be very complex and many factors may have a role in this association such as the obesity [29], level of physical activity [19], serum leptin [30], and other dietary components [29, 30]. However, only overweight subjects were included because of the possible effect of BMI on the association between *FTO* genotype and dietary intake.

Regarding to dietary carbohydrate, the AA genotype carriers had higher carbohydrate intake than TT genotype carriers which was in line with the results of the previous studies [31–33]. Sonest et al. found that *FTO* genetic variants are associated with the amounts of carbohydrate intake. Some study reported that carbohydrate intake (especially glucose intake) increased *FTO* gene expression [31, 32]. In homozygous people for the risk allele of *FTO* gene rs9930506 polymorphism, higher dietary carbohydrate intake had a positive association with *FTO* gene expression [32].

This study found no association between protein intake and *FTO* genotype. While some studies indicated that protein intake was associated with *FTO* genotype [33, 34]. However, another study reported that leucine intake increased *FTO* gene expression [34]. Doaei et al. found that higher protein intake up-regulated the *FTO* gene and also indicated that only in A allele carrier [32].

The mechanism of the interactions between the *FTO* genotype and dietary macronutrients is not fully understood. The *FTO* gene polymorphisms may change the amounts of macronutrients’ intake. On the other hand, the association of *FTO* polymorphisms with obesity may be influenced by dietary intake. It was observed that the A risk allele of *FTO* rs9939609 polymorphism had no significant association with obesity in subjects whose dietary fat intake was below 30% of total energy, but increased central and total adipose tissues in subjects with fat intake higher than 30% [35]. Another study reported that the risk allele carriers who received Mediterranean diet for 3 years had lower BMI compared with the others [36]. Dietary macronutrients may also change the level of *FTO* gene expression. Nowacka-Woszuk et al. indicated that a high-fat diet could increase *FTO* gene expression in white adipose cells in rats [31]. Ronkainen et al. investigated the association between fat intake and the *FTO* gene expression. They found that a high-fat diet could suppress *FTO* expression [32].

Some studies suggested that *FTO* play a crucial role in regulating energy homeostasis. *FTO* gene is expressed in hypothalamus that controls feeding and energy expenditure [36, 37]. Interestingly, *FTO* expression level in hypothalamus is regulated by dietary intake. It was reported that a high-fat diet can down-regulate *FTO* expression in short-term and up regulate it in long-term [38, 39].

On the other hand, the *FTO* gene is related with gut hormones such as orexigenic hormone, acyl-ghrelin, satiety hormone, peptide YY that regulate food intake and appetite [40]. *FTO* gene polymorphism (AA genotype) influence on circulating PYY3–36 and acyl-ghrelin levels that lead to increased food intake especially energy-dense foods and reduced satiety [41, 42]. In rs9939609 AA carriers, suppression of acylated ghrelin led to overeating and obesity [43]. So, it is plausible that *FTO* gene polymorphisms could change appetite and food intake that may lead to weight gain and obesity.

### Study strengths and limitations

The main strength of this study was the relatively high sample size of overweight adults and adjustments for sugar and lipid profiles as the possible factors affecting dietary intake. This study also included only overweight subjects because of the possible effect of BMI on the association between *FTO* genotype and dietary intake. In addition, information on a wide range of potential confounders/modifiers and their potential effects were taken into account. The present study also has several limitations to acknowledge. First, the study was limited by cross-sectional design. Second, dietary intake was determined according to a self-reported questionnaire, this parameter was not measured objectively although similar to many prior epidemiological studies.

## Conclusion

The genotype of *FTO* may influence the amount of dietary intake in overweight people. *FTO* gene rs9939609 polymorphism was associated with dietary intake. The intake of calorie, carbohydrate, and fat intake were associated with *FTO* gene polymorphisms and this association remained significant for calorie and macronutrients after adjustments for sex, age, physical activity, LDL, HDL, and FBS. In AA carriers, dietary carbohydrate, fat, calorie was higher than TT carriers. Genetic profile can play a key role in future nutritional recommendations especially for weight management and also for prevention of diet-related chronic diseases. Diet therapy in people with risk allele of *FTO* rs9939609 polymorphism may require to consider their desire to eat more carbohydrate, fat, and calorie. Further studies are needed to increase understanding of the underlying mechanisms of the association between *FTO* gene and dietary intake.

## Supplementary information

**Additional file 1.**

## Data Availability

Not applicable.
