# Supplementary material for: The association between FTO genotype with macronutrients and calorie intake in overweight adults
Source: Lipids Health Dis. 2020 Aug 26;19:197. doi: 10.1186/s12944-020-01372-x (PMC7449073; doi:10.1186/s12944-020-01372-x)
Supplement: Supplementary file 1 — Additional file 1. [file 12944_2020_1372_MOESM1_ESM.docx]

Supplementary file 1. The primers sequence used to tetra-primer amplification refractory mutation system-polymerase chain reaction (Tetra-ARMS PCR).

| FFTO-Arms | GTGAGGAATACTAGGAGAGGAGAA |
| --- | --- |
| RFTO-N-ARMS | AGAGACTATCCAAGTGCATCAGA |
| RFTO-M-Arms | CAGAGACTATCCAAGTGCATCAAT |
| Sequencing primer | ACAAATGTTCAAGTCACACTCAG |
